# Influence of abiotic factors in Calliphoridae and Mesembrinellidae (Insecta: Diptera) entomofauna in the Jardim Botânico do Rio De Janeiro, Brazil

**DOI:** 10.1371/journal.pone.0322487

**Published:** 2025-05-02

**Authors:** Adriana Leal de Figueiredo, Jéssica da Silva Costa, Wellington Thadeu de Alcantara Azevedo, Maria Lucia França Teixeira, Cláudia Soares Santos Lessa, Valéria Magalhães Aguiar

**Affiliations:** 1 Programa de Pós-Graduação em Biodiversidade Neotropical, Universidade Federal do Estado do Rio de Janeiro, Rio de Janeiro, Brazil; 2 Departamento de Microbiologia e Parasitologia, Universidade Federal do Estado do Rio de Janeiro, Rio de Janeiro, Brazil; 3 Jardim Botânico do Rio de Janeiro, Rio de Janeiro, Brazil; Instituto Leonidas e Maria Deane / Fundacao Oswaldo Cruz, BRAZIL

## Abstract

Diptera are one of the four megadiverse insect orders, with great environmental, ecological, forensic and medical-sanitary relevance. The Jardim Botânico do Rio de Janeiro, located in an urban area of Rio de Janeiro, Brazil, constitutes an important refuge, supporting the conservation of several species. Through the knowledge of the diversity of Calliphoridae and Mesembrinellidae in this location, it will be possible to identify the behavior of native species along with the invasive genus *Chrysomya*, and to evaluate the influence of abiotic factors (temperature, relative humidity and pluviosity) on insect capture. Eight traps containing sardines were set and a total of 36,035 Diptera specimens were collected, of which 35,890 were of the Calliphoridae family and 145 of the Mesembrinellidae family. The average abiotic variables recorded were: Temperature 25,6 ºC (± 3,17), humidity 68% (±9,33%) and rainfall 3.42 mm (± 7,99). The total abundance of Calliphoridae and Mesembrinellidae peaked during January 2015, with the highest temperature. Among the variables and abundance and richness indices, significant results were only observed between temperature and abundance. None of the variables showed significant correlation with Calliphoridae and Mesembrinellidae richness. The presence of some species of Calliphoridae and Mesembrinellidae indicates the area’s good conservation status as they only occur in highly preserved forest areas. Through the knowledge of their richness and abundance, new legislation can be developed to help conservation efforts in deeply modified environments.

## Introduction

Diptera are one of the four megadiverse insect orders (Coleoptera, Hymenoptera, Lepidoptera and Diptera), with great environmental [1], ecological and medical-sanitary relevance due to the fact that some species are carriers of disease-causing pathogens and may cause myiasis, characterized by the presence of fly larvae in human or other animal’s tissues [2,3]. Calliphoridae family comprises mostly of metallic-toned flies, with biontophagous or necrobiophagous habits, commonly attracted to decaying organic matter, and of typically urban, rural or forest habits, with some groups presenting high synanthropy, such as *Chrysomya* sp. [4]. Members of the Mesembrinellidae family are exclusively Neotropical [5]. They are generally robust dipterans, with sizes ranging from 7 to 16 mm, brown colored, and may have metallic coloration in the thorax and abdomen [6]. In Brazil, three genera and nineteen species are currently recorded, which are found only in humid tropical forests [7].

Calliphorids are widely used in forensic entomology to assist in the resolution of judicial and criminal proceedings, especially in cases where it’s necessary to determine the Post-Mortem Interval (PMI) [8,9]. This science, which studies the application of insects in legal proceedings, has been expanding in Brazil, where studies have been carried out using mainly animal carcasses or baits [10–12]. Calliphorids and mesembrinelids are also important pollinators and environmental bioindicators, due to their high sensitivity to changes in the environment [1,13,14].

Several studies have shown the influence of biotic and abiotic factors on insects. The occurrence and abundance of dipteran species have been associated with variations in temperature, humidity and rainfall [15–17]. The importance of abiotic factors is due to the fact that they are possible limiting agents to the Diptera fauna, influencing their development, reproduction and behavior [18]. Among the biotic factors, some authors suggest that the availability of food resources may influence the population dynamics of Diptera [19,20], and such resources are associated with habitat characteristics [21].

The Atlantic Forest is a global *hotspot* of biodiversity and endemism, critically threatened with extinction due to anthropic pressure [22,23]. Its territorial occupation has reduced it to forest fragments, with the remnants of native vegetation reduced to about 29% of its original coverage, preserved in vegetation fragments remnants, given the presence of microclimates and different resource availability, different points in the same fragment may present communities with different richness and diversity [24].

Jardim Botânico do Rio de Janeiro (JBRJ) comprises the arboretum located in an urban area in the southern region of Rio de Janeiro, Brazil, and was founded by D. João VI in 1808 as an acclimatization garden that would serve as a breeding site for oriental plant species, which were used as spices [25]. It constitutes an important urban refuge for fauna and flora, acting as a green space and supporting the conservation of several pollinator species. JBRJ includes a fragment of continuous Atlantic Forest area that is connected to both the Parque Natural Municipal da Cidade and the Parque Nacional da Tijuca (PARNATijuca), very important spots for conservation of this biome [26,27]. The arboretum area is open to public visitation and provides visitors with a very diverse space where about 6,500 plant species, many endangered or exotic, can be observed. The disordered growth of urban areas has caused changes in native environments, causing the emergence of new types of habitats, which are occupied by the species better adapted to the new conditions [18]. There are few studies on dipterofauna surveys in the JBRJ and in the forest fragment near the arboretum [28]. A deeper Calliphoridae and Mesembrinellidae diversity knowledge will allow for native species behavior identification, notably species of the genus *Chrysomya* Robineau-Desvoidy, 1830, such as *Chrysomya megacephala* Fabricius, 1794 and *Chrysomya albiceps* Wiedemann, 1819. These were introduced in Brazil in the 70s, and studies demonstrate changes in local fauna in various environments after its introduction in the American Continent [29]. JBRJ is ana rea of conservation and study of local flora and fauna species, and an important tourist attraction, receiving constant visitation from a large number of people. The identification of Calliphoridae and Mesembrinellidae bioindicator species within the JBRJ will bring important information regarding the location’s environmental health.

Therefore, the present study aimed to analyze the species that compose the dipterofauna of the Calliphoridae and Mesembrinellidae families in the Jardim Botânico do Rio de Janeiro (JBRJ), to evaluate the influence of abiotic factors on insect capture at four collection points, including the Atlantic Forest fragment, and compare collection points at both the preserved and public visitation areas.

## Materials and methods

Eight traps were used to collect the insects following the model of [30], containing about 400g of raw sardines (*Sardinella brasiliensis* Steindachner, 1879) (Teleostei: Dorosomatidae), which were thawed in a refrigerator 24 hours before being exposed, to act as bait. The traps consist of a PVC pipe base where the bait is placed, sealed with a transparent plastic container with an inverted funnel in its interior to catch the insects. All traps were positioned at a height of 1.5 m from the ground. Sardines were purchased from a supermarket near the study site and did not require Institutional Animal Care and Use Committee (IACUC) approval. The collections were carried out monthly, and the traps remained exposed for 48 hours during each month. All insects caught during the study period were taken to the Laboratório de Estudo de Dípteros (LED) of the Departamento de Microbiologia e Parasitologia, Universidade Federal do Estado do Rio de Janeiro (UNIRIO) and identified using the taxonomic keys of [31] and [32], with the updates of [7].

**Fig 1:**
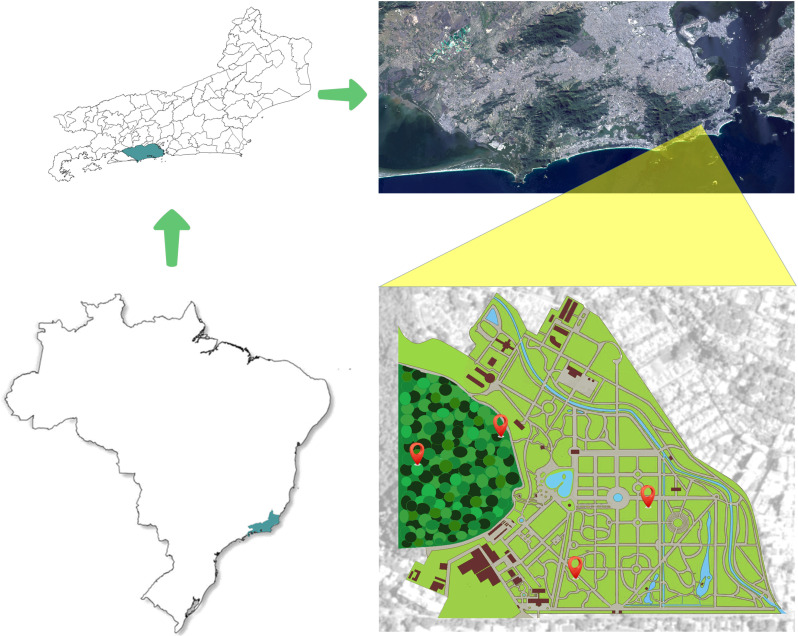
Distribution of the four capture points from July 2014 to June 2015 and delimitation of the Jardim Botânico do Rio de Janeiro.

The research was carried out in the arboretum of the Jardim Botânico do Rio de Janeiro and in a fragment of Atlantic Forest, located in the Jardim Botânico district, Rio de Janeiro, between July/2014 and June/2015. The Jardim Botânico has an area of 140 hectares connected to the adjacent Floresta da Tijuca, of which 54 belong to the arboretum, being in a tropical climate region. Four of the traps were installed in the arboretum area: two next to the street (point A - 22°58’11,26“S 43°13’27,35”W) and two in an inner point in the JBRJ (point B - 22°58’07,48”S 43°13’27,35”W). The remaining traps were installed in the forest fragment, two at the edge of the forest (point C - 22°58’03,85”S 43°13’41,95”W) and two 100 m from the edge (point D - 22°58’03,68”S 43°13’46,34”W) (Fig 1). At each point, the traps were installed about 1.5 m from the ground, five meters apart. The meteorological data of the sites on the days of insect collection were taken from the website of the Instituto Nacional de Meteorologia (INMET), with the closest Meteorological Station from the experimental areas (22°58’22.0”S 43°13’26.0”W). The closest trap was placed 350m, approximately, from the station, while the farthest one was located at around 790m from it.

The abundance and richness of the sampled species were measured, as well as the proportion of males and females. Pearson’s (normal data) and Spearman’s (nonparametric data) correlation tests were performed using the R program to verify whether there was a functional relationship between the abundance and richness of calliphorids and mesembrinelids and the abiotic variables (temperature, relative humidity and rainfall). Correlation classification was based on its values, with 0.0 to 0.2 given as null correlation, 0.21 to 0.40 as weak correlation, 0.41 to 0.70 as substantial correlation, 0.71 to 0.90 as strong correlation, and 0.91 to 1.0 as extremely strong correlations [33].

## Results

**Fig 2:**
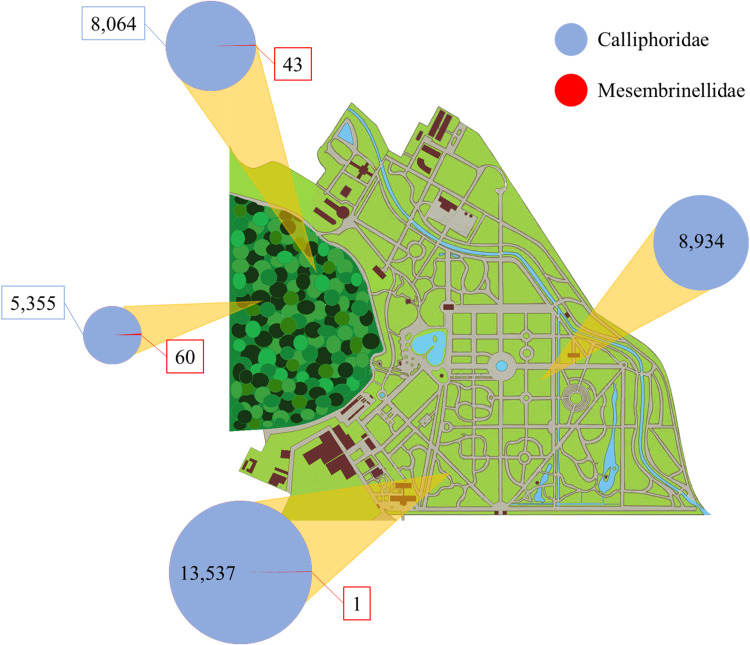
Abundance of Calliphoridae and Mesembrinellidae at the four capture points from July 2014 to June 2015 at Jardim Botânico do Rio de Janeiro.

During the study, a total of 36,035 Diptera specimens were collected during the period the traps were exposed, of which 35,890 were specimens of the Calliphoridae family and 145 of the Mesembrinellidae family, comprising thirteen species of seven genera. The abundance recorded at each point was as follow: At point A, 13,538 Calliphoridae and 1 Mesembrinellidae; 8,934 Calliphoridae at point B; 8,141 Calliphoridae at point C; and 43 Mesembrinellidae and 5,422 Calliphoridae and 60 Mesembrinellidae at point D (Fig 2). Data regarding the correlation between abundance and species richness were published by [28].

Among the collected calliphorids, the most abundant were *C. megacephala* and *C. albiceps*. In addition to these, the following species were identified: *Chloroprocta idioidea* Robineau-Desvoidy 1830, *Chrysomya putoria* Wiedemann 1818, *Cochliomyia hominivorax* Coquerel 1858, *Cochliomyia macellaria* Fabricius 1775, *Hemilucilia segmentaria* Fabricius 1805, *Hemilucilia semidiaphana* Rondani 1850, *Lucilia cuprina* Wiedemann 1830 and *Lucilia eximia* Wiedemann 1819. *Laneella nigripes* Guimarães 1977 and *Mesembrinella bellardiana* Aldrich 1922 were the species collected of the Mesembrinellidae family.

**Fig 3:**
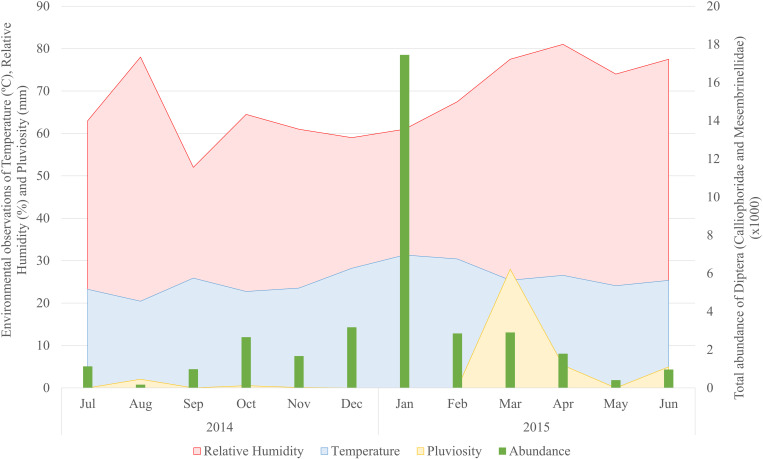
Records of climatic variables during the Diptera capture in the Jardim Botânico do Rio de Janeiro using sardines as bait with monthly collections from July 2014 to June 2015.

The recording of abiotic variables revealed that the temperature during the collection days averaged 25.6 ºC (± 3.17), ranging from 20.45 ºC in August 2014 to 31.35 ºC in January 2015. The average humidity was 68% (± 9.33%), with the highest value recorded in April 2015 (81%) and minimum in September 2014 (52%). Pluviosity was low throughout the period, with an average of 3.42 (± 7.99) mm, with the highest record of 28 mm in April 2015. During most of the experiment no pluviosity was recorded (Fig 3). The total abundance of Calliphoridae and Mesembrinellidae, in contrast to these variables, peaked during January 2015, when the highest mean temperature was registered. On the other hand, during August 2014, the lowest capture rate and the lowest average temperature were documented (Fig 3).

**Fig 4:**
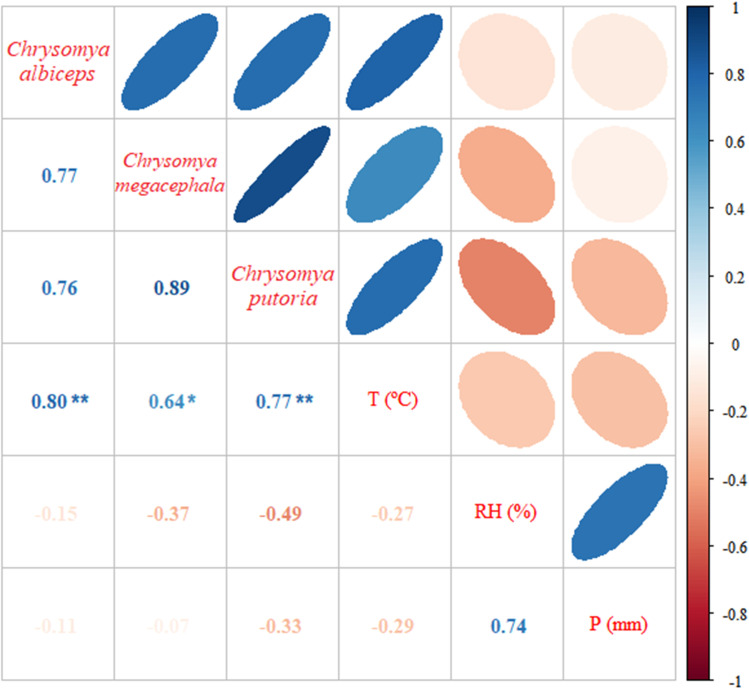
Correlation matrix between climatic variables (T - Temperature, RH - Relative Humidity, P - Rainfall) and Calliphoridae species captured in the Jardim Botânico do Rio de Janeiro from July 2014 to June 2015. Statistically significant correlation can be seen. * Significant to alpha (α) of 5%; ** Significant to alpha (α) of 1%.

Once the normality of the data was confirmed by Shapiro-Wilk’s test, Pearson’s correlation test (normal data) was used for the correlation between *C. putoria*, *L. eximia* and *La. nigripes* with Temperature (T) and Relative Humidity (RH). Spearman’s test (nonparametric data) was used for the other species and correlation tests with rainfall (P). Three Calliphoridae species showed a positive and significant correlation between their abundance and temperature: for the species *C. albiceps* and *C. putoria*, there is a strong positive correlation between its abundance and measured temperature, obtaining correlation indices equal to 0.804 (p = 0.002) and 0.767 (p = 0.004), respectively, while *C. megacephala* showed a positive and substantial correlation between the same variables (rho = 0.636, p = 0.026) (Table 1; Fig 4).

**Table 1:** Correlation indices between abundance of Calliphoridae and Mesembrinellidae species captured in the Jardim Botânico do Rio de Janeiro and the abiotic factors (T - Temperature, RH. – Relative Humidity, P - Rainfall).

Species	T		R.H.		P	
	cor	p	cor	p	cor	p
*Chloroprocta idioidea*	0,116 ^f^	0,720	0,531 ^b^	0,076	0,264 ^c^	0,408
*Chrysomya albiceps*	0,804 ^a^	0,002**	-0,147 ^f^	0,648	-0,108 ^f^	0,738
*Chrysomya megacephala*	0,636 ^b^	0,026*	-0,368 ^e^	0,239	-0,075 ^f^	0,818
*Chrysomya putoria*	0,767 ^a^	0,004**	-0,492^ d^	0,104	-0,328^ e^	0,297
*Cochliomyia hominivorax*	0,462 ^b^	0,130	0,065 ^f^	0,840	-0,054 ^f^	0,868
*Cochliomyia macellaria*	0,147 ^f^	0,648	-0,534 ^d^	0,074	-0,288^ e^	0,365
*Hemilucilia segmentaria*	0,186 ^f^	0,564	0,257 ^c^	0,421	0,239 ^c^	0,436
*Hemilucilia semidiaphana*	-0,028 ^f^	0,931	-0,397 ^e^	0,202	-0,358 ^e^	0,253
*Laneella nigripes*	-0,508 ^d^	0,092	0,574 ^b^	0,051*	0,725 ^a^	0,008**
*Lucilia cuprina*	-0,008 ^f^	0,979	-0,264 ^e^	0,406	-0,196 ^f^	0,541
*Lucilia eximia*	0,190 ^f^	0,554	0,198 ^f^	0,538	0,081 ^f^	0,858
*Mesembrinella bellardiana*	-0,113 ^f^	0,727	0,069 ^f^	0,831	0,117 ^f^	0,718

a – Strong and positive correlation/ b – Substantial positive correlation/ c – Weak and positive correlation/ d – Substantial negative correlation/ e – Weak and negative correlation/ f – Null correlation (following [33]).

Pearson’s test (normal data) was used for the correlation between *Chrysomya putoria*, *Lucilia eximia* and *Laneella nigripes* with Temperature (T) and Relative Humidity (RH). Spearman’s test (nonparametric data) was used for the other species and correlation tests with Rainfall (P).

* Significant to alpha (α) of 5%; ** Significant to alpha (α) of 1%.

**Fig 5:**
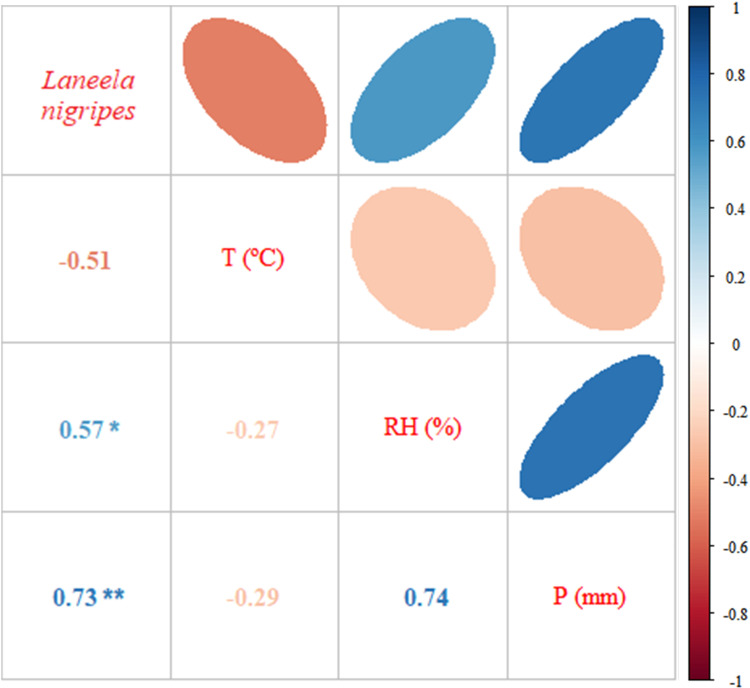
Correlation matrix between climatic variables (T - Temperature, RH - Relative Humidity, P - Rainfall) and *Laneella nigripes* (Diptera: Mesembrinellidae) captured in Jardim Botânico do Rio de Janeiro from July 2014 to June 2015. A statistically significant correlation can be seen. *Significant to alpha (α) of 5%; ** Significant to alpha (α) of 1%.

No species showed positive and strong correlations between abundance and relative humidity, with only *Ch. idioidea* (r = 0.531) and *La. nigripes* (r = 0.574) exhibiting a positive and substantial correlation between species abundance and relative humidity (Fig 4), however, only *La. nigripes* (p = 0.051) showed significant results. *Laneella nigripes* was the sole species to obtain a positive, strong and significant correlation between species abundance and pluviosity (r = 0.725; p = 0.008) (Table 1; Fig 5).

**Fig 6:**
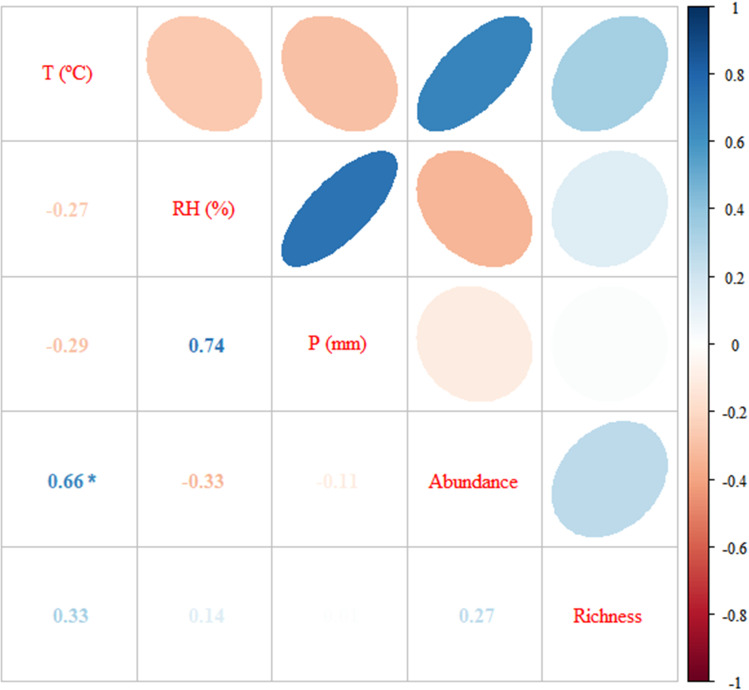
Correlation matrix between climatic variables (T - Temperature, RH - Relative Humidity, P - Rainfall), the abundance and richness of Calliphoridae and Mesembrinellidae species captured in the Jardim Botânico do Rio de Janeiro from July 2014 to June 2015. * Significant to alpha (α) of 5.

Pearson’s test (normal data) was used for the correlation between Richness together with Temperature (T) and Relative Humidity (RH). For abundance and correlation tests with rainfall (P) Spearman’s test (nonparametric data) was used. Among the abiotic variables and along with the abundance and richness indices, correlation showing significant results was only observed between temperature and abundance (positive and substantial correlation by Spearman’s test with a value of 0.664 and p = 0.0185) (Table 2; Fig 6). The correlation between abundance and humidity was negative and weak (r = - 0.333, p = 0.290) and null between abundance and pluviosity (r = - 0.108, p = 0.738), both of which were not significant. The lowest richness occurred in August 2014, when the lowest abundance also occurred, combined with the lowest recorded temperature and a high RH, with a slight increase in rainfall.

**Table 2:** Correlation indices between richness and abundance of Calliphoridae and Mesembrinellidae captured in the Jardim Botânico do Rio de Janeiro from July 2014 to June 2015 and the abiotic factors.

Abiotic factor	Richness		Abundance	
	cor	p	cor	p
Temperature	0,331 ^b^	0,294	0,664 ^a^	0,0185*
Relative Humidity	0,137 ^d^	0671	- 0,333 ^c^	0,290
Pluviosity	0,011 ^d^	0,972	- 0,108 ^d^	0,738

a – Substantial and positive correlation/ b – Weak and positive correlation/ c – Weak and negative correlation/ d – Null correlation (following [33]).

Pearson’s test (normal data) was used for the correlation between Richness with Temperature (T) and Relative Humidity (RH). For abundance and correlation tests with Rainfall (P) spearman’s test (nonparametric data) was used.

* Significant to alpha (α) of 5%.

## Discussion

For this experiment, the choice of sardines as the bait was due to the fact that they have proven to be attractive for members of the Calliphoridae and Mesembrinellidae families, as in the studies of [18,24,34] and [35], and also for their low cost. [36] carried out an experiment under similar conditions to ours, testing different baits to examine their attractiveness to calliphorids, raw sardines among them. Like in the present study, the traps remained exposed during 48 hours, allowing its decomposition. Their study shows that sardines were the most attractive bait for calliphorids, likely due to its strong smell and the fact that they decompose quickly. A high abundance of the *Chrysomya* genus on fish has also been observed in other studies [37–39].

[40] observed the abundance of Calliphoridae in an Atlantic Forest area in Itaboraí (Rio de Janeiro), Brazil, where seven different species were collected, with prevalence of *C. megacephala* during all four seasons, mainly spring and summer. [8] found 16 species distributed in urban and forest areas, with *C. albiceps* being the most abundant species in the area of Atlantic Forest vegetation, in Rio Grande do Sul, Brazil. Similarly, in a more recent study, [17] found 12 species of Calliphoridae distributed in six genera and 4 species of two genera of Mesembrinellidae, with *C. albiceps* presenting as the species of highest abundance, followed by *C. megacephala*, in a fragment of Atlantic Forest near the Paraná River, in Brazil. In a mountainous forest in Germany, [41] observed that calliphorid dipterans were more abundant in traps containing doe (*Capreolus capreolus* Linnaeus, 1758) viscera than in the carcass of the animal itself, possibly due to the presence of exposed blood in the baits, which could be very attractive for adult flies.

Corroborating other studies, both [42] and [9] also observed prevalence of the *C. megacephala* species in Atlantic Forest fragments, collecting, respectively, eight and eleven Calliphoridae species and a low diversity of Mesembrinellidae in Engenheiro Paulo de Frontin, RJ and Recife, PE, in Brazil. [15] hypothesized that environments with a greater complexity such as forests end up having a greater diversity, because they offer a higher number of potential niches, allowing a superior number of species. According to the authors, forests would also provide environments with greater food diversity, shaded sites and more stable environments for the species.

In addition to environmental complexity, the number of captured Diptera is also influenced by abiotic variables (temperature, humidity and rainfall, mainly), with a reduction of populations in the months with milder temperatures [16]. Dipterans depend on relatively high temperatures to develop, with some having a preference for warmer climates for egg laying [43,44]. According to [8], temperature and humidity are among the main regulating factors for calliphorids, with temperature usually being a more important one than humidity. The increase in rainfall, in turn, can hinder the flight of Diptera and make the bait less attractive to them [24]. [45] suggests that each species reacts in its own particular way to environmental conditions and that its population dynamics is not only influenced by climatic factors, but possibly by the presence of several types of substrates created and directly influenced by man.

[46] found that the temperature significantly affected the number of calliphorids caught in traps containing pork in localities with different urbanization rates and also the species found in Connecticut, USA. [15] observed lower richness and capture of Diptera during the winter, when the gauged temperatures were lower, and greater abundance of *Co. macellaria*, *C. putoria*, *C. megacephala* and *C. albiceps* during the warmer months (September to May). [47] noted that the richness in species capture tends to be higher in forest environments than in urban environments in Switzerland. [48] also observed an increase in the number of species during Diptera collection in the months of higher temperature (April, February and March), with *C. megacephala* presenting itself as the most numerous species in a forest area of Niterói, Rio de Janeiro. Likewise, in this study, abundance peaked in January, which was the warmest month, followed by February and April. As a city of tropical climate, the temperatures in Rio de Janeiro remain steady on the higher side, varying between 20–30ºC. Even then, abundance was considered low on during the colder winter months (June, July and August).

However, [49] reported a higher population abundance of *Chrysomya* species in the months with temperatures between 18.5°C and 23.5°C, and in the warmer months, the abundance was significantly lower in Pelotas, Rio Grande do Sul, Brazil. In contrast to the present study, [24] observed no correlation between abundance of Calliphoridae and Mesembrinellidae and temperature in a study conducted in the Reserva Biológica do Tinguá, RJ.

According to [50], *H. semidiaphana* prefers warmer temperatures, however, in this study, the species showed null correlation between its abundance and temperature. We hypothesize that *H. semidiaphana* may be able to tolerate variations in temperature since other studies have encountered the species in environments which tend to present different conditions such as urban settlements, forest and rural areas [24,51,52]. [34] also observed an increase in the abundance of *C. megacephala* in the warmer months of the year and attested a strong correlation between the *C. albiceps* abundance and the temperature. [53] recorded a drop in *C. albiceps* capture during a period of high temperatures and low pluviosity in South Africa. Likewise, [54] noted the species’ preference for mild and warm temperatures. In northern Thailand, [55] found a moderately positive correlation between temperature and abundance of *C. megacephala* and moderately negative correlation between its abundance and relative humidity, with a peak of abundance during the summer, noting that variations in abiotic factors such as temperature and humidity have a significant impact on the species’ abundance and its seasonal fluctuation. According to the findings of [56], however, there is a negative correlation between the abundance of *C. megacephala*, temperature and relative humidity.

For [57], exotic species have high tolerance to climatic variations (temperature, relative humidity and luminosity), which can be considered as a determining factor for high adaptive capacity and a facilitator for geographic expansion. [35] observed that the invasive species *C. megacephala* and *C. albiceps* were more abundant in periods of lower humidity and higher heat, while [17] found a correlation between temperature and the presence of both species. Like other ectotherms, flies possess a temperature requirement for full development, and different strains may present different requirements. Both *C. putoria* and *C. albiceps* populations found in Brazil proceed from a tropical climate area in the African continent, therefore, would be well adapted to higher temperatures [58]. The species have also shown a faster developmental rate when in higher temperatures [59,60].

For [40], *Ch. idioidea* can be considered well adapted to the four seasons, but has a preference for colder seasons. The species was found to be the most abundant in the Andean Amazon region of Colombia during the dry season [61]. [24] did not observe a positive correlation of *Ch. idioidea* with any environmental variable, corroborating our results. Regarding *La. nigripes*, [34] observed a strong correlation with temperature, but not with humidity, differing from the present study which reinforces that some species may present differing behaviors in distinct environments. We hypothesize that the species rainfall’s strong correlation may be related to its preference for forest habitats, as these environments are usually associated with a high humidity and pluviosity in tropical climates such as the one in this study. More studies would be necessary to explain the reason behind this, as Mesembrinellidae remains a family with a rather small number of studies. [62] found moderate correlation between abundance and rainfall for *L. eximia*, but not significant, while [56] noted no significant correlation between the number of captured calliphorids and the rainfall index.

[24] and [34] found no correlation of Calliphoridae and Mesembrinellidae abundance with humidity or rainfall in Rio de Janeiro. In a study in Thailand, humidity also showed no correlation or influence on the abundance of species belonging to the genus *Chrysomya*. [63]. [64], however, observed a decrease in the number of insects caught in traps containing pork baits during rainy days in an Atlantic Forest area in São Paulo, Brazil. [65] found a correlation between climatic variables such as rainfall, temperature and cloudiness and the abundance of calliphorids belonging to the genus *Lucilia* Robineau-Desvoidy, 1830, when conducting an experiment to collect members of the Calliphoridae family in a forest area in Argentina. [66] observed different patterns between abiotic variables and the abundance of Mesembrinellidae at the Reserva Biológica do Tinguá, with null correlation between abundance and rainfall and weak and negative between species richness and rainfall. There are few studies involving ecology and taxonomic identification of mesembrinelids, making it difficult to analyze the results presented here in relation to this family.

The Diptera insects of the Calliphoridae family can be found in urban, rural and forest regions, thus its correct identification is crucial. A few of the species found are known producers of myiasis in both humans and animals, inflicting great losses and affecting animals. JBRJ acts as an urban refuge for the local fauna, which is very rich and diverse, hence the need for a deeper knowledge of possible hazards. Although it is speculated by some authors that, due to its similar necrophagous habits, Mesembrinellidae may also pose harm to the public health and serve as a tool for calculating the PMI in forensic entomology, such claim needs to be further investigated and tested for corroboration as there are few evidence that may indicate that they can be used for such. Knowledge about the family distribution can be a useful tool for environmental entomology, as its species can be considered bioindicators of the conservation status of an area due to its preference for well-preserved environments [67].

## Conclusion

Regarding both groups richness, 13 species belonging to four subfamilies, Chrysomyinae, Luciliinae, Mesembrinellinae and Laneellinae, were found composing the Calliphoridae and Mesembrinellidae fauna in JBRJ. Four of the species catalogued showed a positive correlation between their abundance and temperature, including all species of the genus *Chrysomya* (*C. albiceps*, *C. megacephala*, *C. putoria*) and *Cochliomyia hominivorax*, while *Laneella nigripes* showed a substantial negative correlation with temperature. Regarding the relative air humidity, only *La. nigripes* showed significant positive and substantial correlation, seemingly reinforcing its status as a species from forest environments, which tend to be moist, and two species showed a negative and substantial correlation (*C. putoria* and *C. macellaria*). A single species showed strong positive correlation with rainfall (*La. nigripes*). The highest abundance of Calliphoridae and Mesembrinellidae was recorded in January 2015, when the highest temperature values were recorded, while August 2014 recorded the lowest abundance, during the lowest temperatures. None of the abiotic variables studied here showed significant correlation with the richness of Calliphoridae and Mesembrinellidae. The presence of Mesembrinellidae species in the locality indicate the preservation status of JBRJ, as these species occur in well preserved forest areas. Through both Calliphoridae and Mesembrinellidae’s richness and abundance, new measures can be developed to better understand and mitigate human impacts, helping conservation efforts in deeply modified environments.
